# On the Stability of MHD Boundary Layer Flow over a Stretching/Shrinking Wedge

**DOI:** 10.1038/s41598-018-31777-9

**Published:** 2018-09-11

**Authors:** Izyan Syazana Awaludin, Anuar Ishak, Ioan Pop

**Affiliations:** 1 Faculty of Management, Multimedia University, 63100 Cyberjaya, Selangor Malaysia; 2 School of Mathematical Sciences, Faculty of Science and Technology, Universiti Kebangsaan Malaysia, 43600 UKM Bangi, Selangor Malaysia; 3 Department of Mathematics, Babeş-Bolyai University, 400084 Cluj-Napoca, Romania

## Abstract

The steady two dimensional magnetohydrodynamic (MHD) boundary layer flow and heat transfer over a stretching/shrinking permeable wedge is numerically investigated. The partial differential equations governing the flow and heat transfer are transformed into a system of ordinary differential equations using a similarity transformation. These equations are then solved numerically using the boundary value problem solver, bvp4c in Matlab software. It is found that dual solutions exist for a certain range of the shrinking strength. A stability analysis is performed to identify which solution is stable and physically reliable.

## Introduction

One of the family of boundary layer similarity solutions was discovered by Falkner and Skan^1^, for the study of flow over a static wedge. The numerical solutions were then calculated by Hartree^2^. With a similarity transformation, the boundary layer equation reduces to an ordinary differential equation, which is well known as the Falkner-Skan equation. This problem reduces to the classical Blasius flow if the angle of the wedge is set to zero, while it reduces to the stagnation-point flow (Hiemenz flow) if the angle is 180°. The Falkner-Skan flow past a stretching wedge was considered by Postelnicu and Pop^3^ and Su *et al*.^4^, while very recently Nadeem *et al*.^5^ studied the Falkner-Skan problem for a static and moving wedge in the presence of induced magnetic field.

Magnetohydrodynamic (MHD) flow is the study of magnetic properties on electrically conducting fluids. Given its significance in engineering industrial applications, this has led to numerous studies of MHD flow in electrically conducting fluids, such as Ishak^6^, Jafar *et al*.^7^, Mat Yasin *et al*.^8^, Hayat *et al*.^9,10^, Lu *et al*.^11^, etc. Dual solutions were found for the unsteady flow over a shrinking sheet by Soid *et al*.^12^. Meanwhile, Sharma *et al*.^13^ in their study of two dimensional MHD stagnation point flow over a stretching/shrinking sheet found dual solutions exist for a certain range of the shrinking parameter. The stability analysis revealed that the first solution is stable while the second solution is not.

With that, the present study examines the steady boundary layer flow and heat transfer over a stretching/shrinking wedge immersed in a viscous and incompressible electrically conducting fluid, with uniform surface temperature. The analysis is conducted numerically by using bvp4c function and once the dual solutions are obtained, the stability analysis is performed to determine which solution is stable and physically reliable.

## Mathematical Formulation

**Figure 1:**
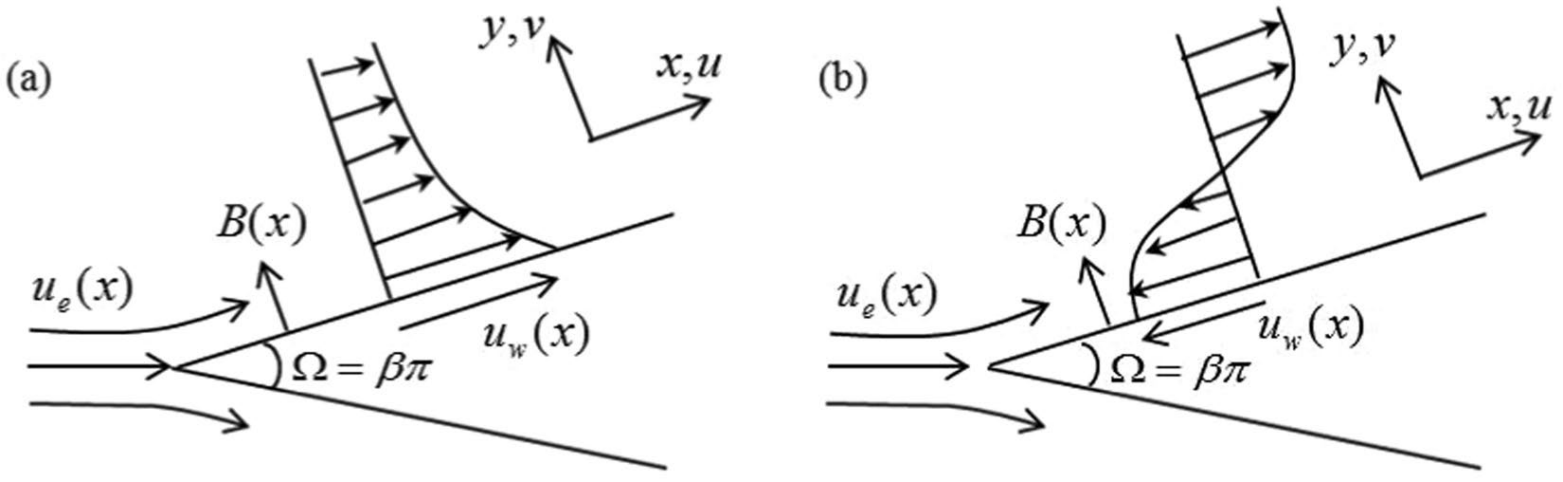
Physical model and coordinate system for (**a**) stretching and (**b**) shrinking wedge.

Figure 1 depicts the physical model and coordinate system for the stretching/shrinking wedge, where *x* and *y* are respectively the Cartesian coordinates measured along the surface and normal to it, while *u* and *v* are the velocity components along the Cartesian coordinates *x* and *y*, respectively. It is assumed that the velocity of the stretching/shrinking wedge is represented by ${u}_{w}(x)={U}_{w}{x}^{m}$, where ${U}_{w} > 0$ corresponds to stretching and ${U}_{w} < 0$ corresponds to shrinking. It is also assumed that the velocity of the external flow outside the boundary layer is represented by ${u}_{e}(x)={U}_{\infty }{x}^{m}$, where *m* and *U*_∞_ are positive constants. A variable magnetic field of strength represented by *B*(*x*) is applied in the positive direction of *y*-axis. The induced magnetic field is assumed small and neglected. In Fig. 1, *β* is the Hartree pressure gradient parameter which corresponds to *β* = Ω/*π* for a total angle Ω of the wedge. We note that $0\le m\le 1$ with *m* = 0 for the boundary-layer flow over a stationary flat plate (Blasius problem) and *m* = 1 for the flow near the stagnation point on an infinite wall.

The governing equations based on the above-mentioned assumptions are (Ishak *et al*.^14^)

1 $$\frac{\partial u}{\partial x}+\frac{\partial v}{\partial y}=0$$

2 $$u\frac{\partial u}{\partial x}+v\frac{\partial u}{\partial y}={u}_{e}\frac{d{u}_{e}}{dx}+\nu \frac{{\partial }^{2}u}{\partial {y}^{2}}-\frac{\sigma {B}^{2}(x)}{\rho }(u-{u}_{e})$$

3 $$u\frac{\partial T}{\partial x}+v\frac{\partial T}{\partial y}=\alpha \frac{{\partial }^{2}T}{\partial {y}^{2}}$$

while the boundary conditions are

4 $$\begin{array}{c}v={v}_{w},\,u={u}_{w}(x),\,T={T}_{w}\,{\rm{at}}\,y=0\\ u\to {u}_{e}(x),\,T\to {T}_{\infty }\,{\rm{as}}\,y\to \infty \end{array}$$

where *ν* is the kinematic viscosity, *T* is the fluid temperature, *T*_*w*_ is the uniform surface temperature, *σ* is the electrical conductivity, *α* is the thermal diffusivity of the fluid, *ρ* is the fluid density and *v*_*w*_(*x*) is the mass flux velocity with ${v}_{w}(x) < 0$ for suction and ${v}_{w}(x) > 0$ for injection.

To obtain similarity solutions of equations (1) to (3) subject to boundary conditions (4), we introduce the following similarity variables (Ishak *et al*.^15,16^):

5 $$\psi =\sqrt{\frac{2\nu x{u}_{e}(x)}{1+m}}f(\eta ),\,\eta =\sqrt{\frac{(1+m){u}_{e}(x)}{2\nu x}}y,\,\theta (\eta )=\frac{T-{T}_{\infty }}{{T}_{w}-{T}_{\infty }}$$

where *ψ* is the stream function, which is defined as *u* = ∂*ψ*/∂*y* and *v* = −∂*ψ*/∂*x* which satisfies the continuity equation (1). Thus, resulting to:

6 $$u={u}_{e}(x)f^{\prime} (\eta ),\,v=-\sqrt{\frac{(1+m)\nu {u}_{e}(x)}{2x}}[f(\eta )+\frac{m-1}{m+1}\eta \,f^{\prime} (\eta )]$$

where prime denotes differentiation with respect to *η*. At the boundary where *η* = 0, the transpiration rate is given by

7 $${v}_{w}=-\sqrt{\frac{(1+m)\nu {u}_{e}(x)}{2x}}s$$

where *s* = *f*(0), a constant parameter with *s* > 0 for suction and *s* < 0 for injection. To obtain similarity solution, all parameters must be constant. For this purpose, we take $B(x)={B}_{0}{x}^{(m-1)/2}$, where *B*_0_ is a positive constant (see Kudenatti *et al*.^17^).

Substituting equations (5) and (6) into equations (2) and (3), the following ordinary (similarity) differential equations are obtained:

8 $${f}^{{\rm{^{\prime} }}{\rm{^{\prime} }}{\rm{^{\prime} }}}+f{f}^{{\rm{^{\prime} }}{\rm{^{\prime} }}}+\beta (1-f{{}^{{\rm{^{\prime} }}}}^{2})+{M}^{2}(1-{f}^{{\rm{^{\prime} }}})=0$$

9 $$\frac{1}{\Pr }\theta ^{\prime\prime} +f\theta ^{\prime} =0$$

which is subject to the boundary conditions:

10 $$\begin{array}{c}f(0)=s,\,f^{\prime} (0)=\lambda ,\,\theta (0)=1,\\ f^{\prime} (\eta )\to 1,\,\theta (\eta )\to 0\,{\rm{as}}\,\eta \to \infty \end{array}$$

where *λ* is the stretching/shrinking parameter, *β* is the pressure gradient parameter, *M* is the magnetic parameter (Hartmann number), *Pr* is the Prandtl number and *s* is the suction/injection parameter, which are defined as:

11 $$\lambda =\frac{{U}_{w}}{{U}_{\infty }},\,\beta =\frac{2m}{m+1},\,M=\sqrt{\frac{2\sigma }{(m+1)\rho {U}_{\infty }}}{B}_{0},\,{\rm{\Pr }}=\frac{\nu }{\alpha }$$

where $\lambda  > 0$ refers to the stretching and $\lambda  < 0$ is for the shrinking case. Notably, it should be mentioned that in the absent of magnetic field (*M* = 0), Eq. (8) reduces to the classical Blasius equation when *β* = 0 and it reduces to the classical Hiemenz equation when *β* = 1.

The physical quantities of interest in this study are the skin friction coefficient *C*_*f*_ and the local Nusselt number $N{u}_{x}$ which can be expressed as:

12 $${C}_{f}=\frac{{\tau }_{w}}{\rho {u}_{e}^{2}(x)},\,N{u}_{x}=\frac{x{q}_{w}}{k({T}_{w}-{T}_{\infty })}$$

where *τ*_*w*_ is the wall shear stress along the stretching/shrinking surface and *q*_*w*_ is the surface heat flux, which are defined as:

13 $${\tau }_{w}=\mu {(\frac{\partial u}{\partial y})}_{y=0},\,{q}_{w}=-\,k{(\frac{\partial T}{\partial y})}_{y=0}.$$

Substituting (6) into (13), one obtains

14 $${{\rm{Re}}}_{x}^{1/2}{C}_{f}={(\frac{1+m}{2})}^{\frac{1}{2}}f^{\prime\prime} (0),\,{{\rm{Re}}}_{x}^{-1/2}N{u}_{x}=-{(\frac{1+m}{2})}^{\frac{1}{2}}\theta ^{\prime} (0)$$

where Re_*x*_ = *u*_*e*_(*x*)*x*/*ν* is the local Reynolds number.

## Results and Discussions

In this study, the nonlinear ordinary differential equations (8) and (9) subject to the boundary conditions (10) were solved numerically using the bvp4c function available in Matlab software (see Shampine *et al*.^18^). The relative error tolerance was set to10^−7^ with the configured convergence criterion to achieve an accuracy of six decimal places. Dual solutions exist in the current study. These solutions are obtained by using different initial guesses for the values of *f* ″(0) and *θ*′(0) where all of the velocity and temperature profiles satisfy the far field boundary conditions (equation 10) asymptotically. For this purpose, we need to set different values of the boundary layer thickness, *η*_∞_. The range of *η*_∞_ for the first solution is between 10 and 20 while for the second solution is between 30 and 40. The initial guesses and the boundary layer thickness vary for different values of parameters. In the numerical computations, we have to make sure that all profiles satisfy the infinity boundary conditions asymptotically, otherwise the numerical results are not valid. Besides, we compare our results with previously published one by other researchers.

Table 1 shows the comparison of numerical results of the skin friction coefficient, *f* ″(0) with those reported by White^19^, Ishak *et al*.^14^ and Postelnicu and Pop^3^ for the case when *λ* = 0 (fixed surface), *M* = 0 (magnetic field is absent) and *s* = 0 (impermeable surface), which shows a favorable agreement. This gives confidence to the other numerical results that reported in the present study. For the sake of brevity, the value of Prandtl numer, *Pr* was set as 1 (such as ionized gases) and magnetic parameter, *M* was set as 0.2.

**Table 1:** Comparison of numerical results of the skin friction coefficient, *f* ″(0)when *λ* = 0, *s* = 0 and *M* = 0 for different values of *β*.

$\beta$	*M*	Skin friction coefficient, *f″*(0)			
		White^19^ (Blasius)	Ishak *et al*.^14^	Postelnicu and Pop^3^	Present study (Numerical results)
0	0	0.4696	0.4696	0.4696	0.469600
0.5	0	—	—	—	0.927680
0.7	0	—	—	—	1.059808
1	0	—	1.2326	1.23259	1.232587

**Figure 2:**
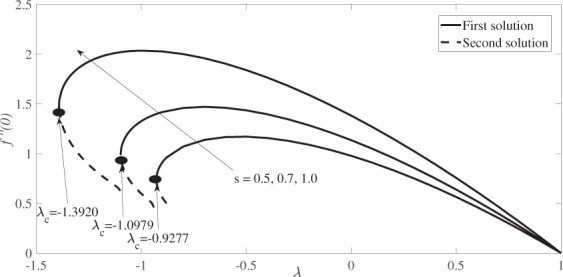
Variation of the skin friction coefficient, *f* ″(0) versus *λ* for various values of *s* when *β* = 0.1 and *M* = 0.2.

**Figure 3:**
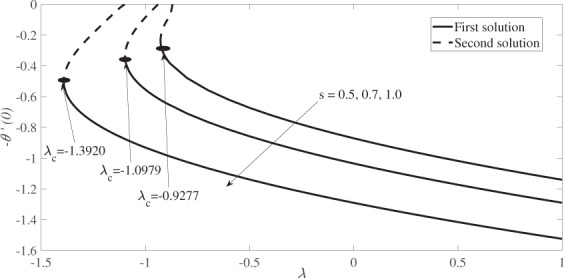
Variation of the local Nusselt number, ***−****θ*′(0) versus *λ* for various values of *s* when *β* = 0.1, *M* = 0.2 and *Pr* = 1.

Figure 2 depicts the variation of the skin friction coefficient, *f* ′′(0) versus *λ* while Fig. 3 depicts the variation of the local Nusselt number, −*θ*′(0) versus *λ*, where $\lambda  < 0$ denotes the shrinking case and $\lambda  > 0$ denotes the stretching case. Variations illustrated in both figures are for different values of *s*, namely *s* = 0.5, 0.7 and 1 at constant values of *β* and *M*, i.e. *β* = 0.1 and *M* = 0.2. Both Figs 2 and 3 reveal the existence of dual solutions (first and second solutions) of equations (8) and (9), subject to the boundary conditions, Eq. (10), for a certain range of *λ* which depends on the suction strength, *s*. The solutions bifurcate at *λ* = *λ*_*c*_, where *λ*_*c*_ is the critical value of *λ* for which equations (8–10) have no solutions for $\lambda  < {\lambda }_{c}$. The values of *λ*_*c*_ are given in Figs 2 and 3. It is observed that dual solutions are found for the shrinking case, while for the stretching case, the solution is unique. Figure 2 also shows that the skin friction coefficient *f* ″(0) increases when the suction strength is increased. Meanwhile, Fig. 3 reveals that the local Nusselt number, −*θ*′(0) which represents the heat transfer rate at the surface, decreases as the effect of suction increases.

**Figure 4:**
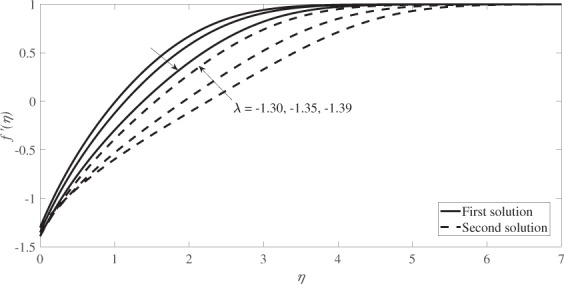
Velocity profiles for several values of *λ* when *s* = 1, *β* = 0.1 and *M* = 0.2.

**Figure 5:**
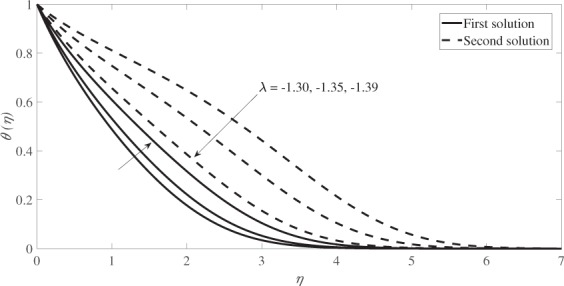
Temperature profiles for several values of *λ* when *s* = 1, *β* = 0.1, *M* = 0.2 and *Pr* = 1.

Figure 4 illustrates the velocity profiles, *f* ′(*η*) while Fig. 5 illustrates the temperature profiles, *θ*(*η*) for different values of *λ* when *s* = 1, *β* = 0.1 and *M* = 0.2. Based on these figures, the boundary layer thickness for the first solution is lower than that of the second solution and the infinity boundary conditions (*f* → 1 and *θ* → 0 as *η* → ∞) are satisfied asymptotically which support the validity of the numerical results obtained.

**Figure 6:**
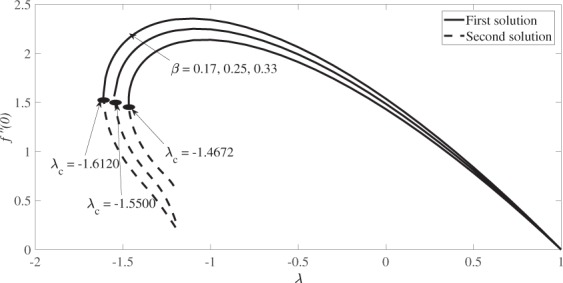
Variation of the skin friction coefficient, *f″*(0) versus *λ* for various values of *β* when *s* = 1 and *M* = 0.2.

**Figure 7:**
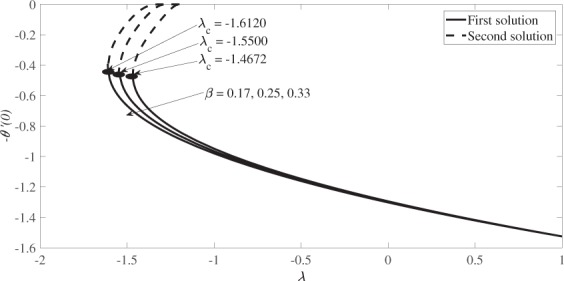
Variation of the local Nusselt number, -*θ′*(0) versus *λ* for various values of *β* when *s* = 1, *M* = 0.2 and *Pr* = 1.

Figure 6 demonstrates the variation of the skin friction coefficient, *f* ″(0) against the stretching/shrinking parameter *λ* for *β* = 0.17, 0.25 and 0.33 when *s* = 1 and *M* = 0.2, while Fig. 7 demonstrates the variation of the associated local Nusselt number, −*θ*′(0) for *Pr* = 1. The numerical results presented in these figures show that dual solutions are obtained for a certain range of the shrinking strength, while the solution is unique for the stretching case. Also, there is no solution for $\lambda  < {\lambda }_{c}$ where these values of *λ*_*c*_ are presented in Figs 6 and 7. These values of *λ*_*c*_ increase in absolute sense, when the values of *β* are increased. Thus, the range of *λ* (for which the solution exists) increases as the angle of the wedge increases. Besides that, the results also indicate that the skin friction coefficient *f* ″(0) increases as *β* increases for the first solutions, whereas it decreases for the second solution. Similar behaviour is observed for the absolute local Nusselt number, |−*θ*′(0)| as presented in Fig. 7.

**Figure 8:**
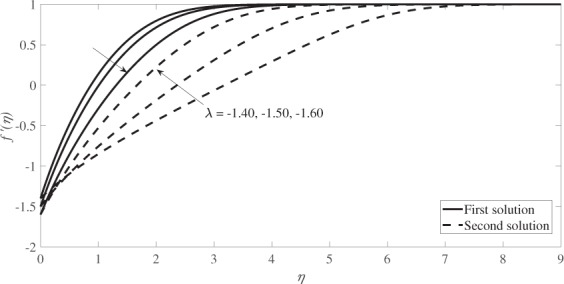
Velocity profiles for several values of *λ* when *β* = 0.33, *s* = 1 and *M* = 0.2.

**Figure 9:**
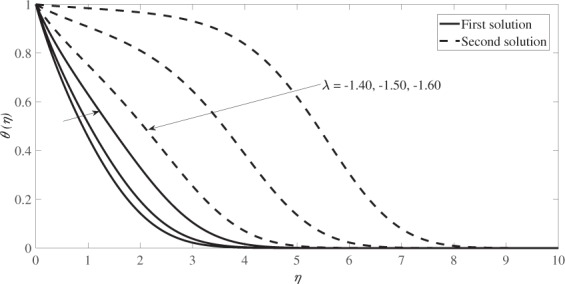
Temperature profiles for several values of *λ* when *β* = 0.33, *s* = 1, *M* = 0.2 and *Pr* = 1.

The graphical results for the velocity profiles *f* ′(*η*) are presented in Fig. 8, while Fig. 9 graphically illustrates the temperature profiles *θ*(*η*) for different values of *λ* for *β* = 0.33, *s* = 1, *M* = 0.2 and *Pr* = 1. Both profiles reveal that the boundary layer thickness for the second solution is higher than that of the first solution and both satisfy the infinity boundary conditions asymptotically, which support the validity of the numerical results obtained.

### Stability Analysis

Since the solutions are not unique for a certain range of the shrinking strength, a stability analysis is performed to determine which one of the solutions is stable and thus physically reliable. There have been several studies on stability analysis such as Merkin^20^, Weidman *et al*.^21^, Roşca and Pop^22^, Merril *et al*.^23^ and Awaludin *et al*.^24^. An analysis of the temporal stability is performed to identify which solution is stable when time passes. The new governing equations for the unsteady state flow are given as

15 $$\frac{\partial u}{\partial t}+u\frac{\partial u}{\partial x}+v\frac{\partial u}{\partial y}={u}_{e}\frac{d{u}_{e}}{dx}+\nu \frac{{\partial }^{2}u}{\partial {y}^{2}}-\frac{\sigma {B}^{2}(x)}{\rho }(u-{u}_{e})$$

16 $$\frac{\partial T}{\partial t}+u\frac{\partial T}{\partial x}+v\frac{\partial T}{\partial y}=\alpha \frac{{\partial }^{2}T}{\partial {y}^{2}}$$

where *t* denotes the time while equation (1) holds. The new similarity variables are introduced as

17 $$\begin{array}{rcl}\psi  & = & \sqrt{\frac{2\nu x{u}_{e}(x)}{1+m}}f(\eta ,\tau ),\,\theta (\eta ,\tau )=\frac{T-{T}_{\infty }}{{T}_{w}-{T}_{\infty }},\\ \eta  & = & \sqrt{\frac{(1+m){u}_{e}(x)}{2\nu x}}y,\,\tau =\frac{1+m}{2}(\frac{{u}_{e}(x)}{x})t\end{array}$$

where *τ* denotes dimensionless time.

Substituting equation (17) into equations (15) and (16) yields

18 $$\frac{{\partial }^{3}f}{\partial {\eta }^{3}}+f\frac{{\partial }^{2}f}{\partial {\eta }^{2}}+\beta [1-{(\frac{\partial f}{\partial \eta })}^{2}]+{M}^{2}[1-\frac{\partial f}{\partial \eta }]-\frac{{\partial }^{2}f}{\partial \eta \partial \tau }=0$$

19 $$\frac{1}{{\rm{\Pr }}}\frac{{\partial }^{2}\theta }{\partial {\eta }^{2}}+f\frac{\partial \theta }{\partial \eta }-\frac{\partial \theta }{\partial \tau }=0$$

which is subject to the boundary conditions:

20 $$\begin{array}{c}f(0,\,\tau )=s,\,\frac{\partial f}{\partial \eta }(0,\,\tau )=\lambda ,\,\theta (0,\tau )=1,\\ \frac{\partial f}{\partial \eta }(\eta ,\,\tau )\to 1,\,\theta (\eta ,\,\tau )\to 0\,{\rm{as}}\,\eta \to \infty .\end{array}$$

To test the stability of the steady flow solution *f*(*η*) = *f*_0_(*η*) and *θ*(*η*) = *θ*_0_(*η*) of equations (8) to (10) we write (see Merril *et al*.^23^):

21 $$\begin{array}{c}f(\eta ,\,\tau )={f}_{0}(\eta )+{e}^{-\gamma \tau }F(\eta ),\\ \theta (\eta ,\,\tau )={\theta }_{0}(\eta )+{e}^{-\gamma \tau }G(\eta ),\end{array}$$

where *γ* is an unknown eigenvalue; *F*(*η*) and *G*(*η*) are small relative to *f*_0_(*η*) and *θ*_0_(*η*). We take perturbation in exponential form since it will increase or decrease more rapidly compared to the power functions. This form of perturbation has also been considered by Merkin^20^, Weidman *et al*.^21^, Roşca and Pop^22^, Merril *et al*.^23^ and Awaludin *et al*.^24^, among others.

Equations (18) to (20) present an infinite set of eigenvalues, ${\gamma }_{1} < {\gamma }_{2} < \mathrm{...}$ . If the smallest eigenvalue is positive, there is an initial decay which is the flow is stable. Besides, there is an initial growth of disturbance if the smallest eigenvalue is negative which mean the flow is unstable. Substituting equation (21) into equations (18) and (19), the following linear eigenvalue problems are obtained:

22 $${F}^{{\rm{^{\prime} }}{\rm{^{\prime} }}{\rm{^{\prime} }}}+{f}_{0}{F}^{{\rm{^{\prime} }}{\rm{^{\prime} }}}+{{f}^{{\rm{^{\prime} }}{\rm{^{\prime} }}}}_{0}F-(2\beta {{f}^{{\rm{^{\prime} }}}}_{0}+{M}^{2}-\gamma ){F}^{{\rm{^{\prime} }}}=0$$

23 $$\frac{1}{Pr}{G}^{{\rm{^{\prime} }}{\rm{^{\prime} }}}+{f}_{0}{G}^{{\rm{^{\prime} }}}+{{\theta }^{{\rm{^{\prime} }}}}_{0}F+\gamma G=0$$

along with the boundary conditions:

24 $$\begin{array}{c}F(0)=0,\,{F}^{{\rm{^{\prime} }}}(0)=0,\,G(0)=0,\\ {F}^{{\rm{^{\prime} }}}(\eta )\to 0,\,G(\eta )\to 0\,{\rm{a}}{\rm{s}}\,\eta \to {\rm{\infty }}.\end{array}$$

**Figure 10:**
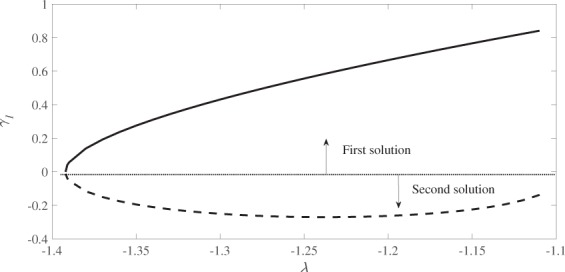
Plot of the lowest eigenvalues ***γ***_***1***_ versus *λ* for *s* = 1, *β* = 0.1 and *M* = 0.2.

The stability of the steady state flow are determined by the smallest eigenvalue *γ*_1_. Since equations (22) and (23) satisfy homogeneous boundary and far-field conditions (24), without loss of generality we set *F*″(0) = 1 to determine the eigenvalues *γ*. There are more that one value of *γ* in equations (22) and (23) that give *F*″(0) = 1. A search for the lowest eigenvalues *γ*_1_ satisfying equations (22) to (24) was carried out and the results are plotted in Fig. 10.

Based on equation (21), the unsteady solution *f*(*η*, *τ*) converges to the steady state solution *F*(*η*) as time passes (*τ* → ∞) if *γ*_1_ is positive. The sign of *γ*_1_ determines either there is an initial decay or initial growth of disturbance. Figure 10 shows that *γ*_1_ is positive for the first solution, and negative for the second solution. Thus, the first solution is stable and physically reliable, whereas the second solution is unstable.

## Conclusions

This study examined the steady two dimensional MHD flow and heat transfer of an incompressible and electrically conducting fluid over a permeable stretching/shrinking wedge. Numerical results, which were obtained using the bvp4c function in Matlab software, proved the existence of dual solutions for the shrinking case but unique solution for the stretching case. For the shrinking case, the existence of the solution depends on the shrinking strength *λ* and the angle of the wedge Ω. The range of *λ* (for which the solution exists) increases as the angle of the wedge Ω increases. Discussion on the skin friction coefficient and the local Nusselt number were carried out for the effect of all parameters involved. The boundary layer thickness for the second solution was found to be higher than that of the first solution, which gave a picture of its stability. To confirm this observation, a stability analysis was conducted and it was revealed that the first solution is stable and physically reliable while the second solution is not.
